# The proteasome regulator PTRE1 contributes to the turnover of SNC1 immune receptor

**DOI:** 10.1111/mpp.12855

**Published:** 2019-08-08

**Authors:** Karen Thulasi Devendrakumar, Charles Copeland, Xin Li

**Affiliations:** ^1^ Michael Smith Laboratories and Department of Botany University of British Columbia Vancouver BC V6T 1Z4 Canada; ^2^ Present address: Department of Plant Microbe Interactions Max Planck Institute for Plant Breeding Research 50829 Cologne Germany

**Keywords:** NLR, plant immunity, proteasome, protein homeostasis, PTRE1, SNC1

## Abstract

Plants have evolved a sophisticated immune system in order to recognize and respond to microbes in their environments. Nucleotide‐binding leucine‐rich repeat (NLR) proteins detect the presence of specific effector molecules delivered into host cells by pathogens and activate strong defence responses. However, as excessive accumulation of NLRs can result in inappropriate immune responses, their abundance must be tightly regulated. Targeted degradation of NLRs through the ubiquitin proteasome pathway is an important mechanism to limit NLR accumulation. Mutations that perturb NLR degradation can cause autoimmune phenotypes. In this study, we show that the proteasome regulator PTRE1 also contributes to NLR degradation. *ptre1* mutant plants exhibit increased defence marker gene expression and enhanced disease resistance against virulent pathogens. The stability of the NLR, SUPPRESSOR OF *npr1‐1* CONSTITUTIVE 1 (SNC1) is also increased in the *ptre1* mutant. Although the mouse homologue of PTRE1 was reported to interact with a Cell Division Control protein 48 (CDC48) homologue *in vitro* (Clemen *et al*., 2015), we only observed interaction between PTRE1 and AtCDC48A in a split luciferase assay, but not in co‐immunoprecipitation. In addition, a related *Arabidopsis* protein PTRE1h shares partial redundancy with PTRE1. Together, PTRE1 acts as a negative regulator of plant immunity partly by facilitating the degradation of immune receptors such as SNC1.

Like all other multicellular organisms, plants face constant threats from pathogens such as bacteria and fungi in their environment. However, they have evolved a sophisticated innate immune system that enables them to recognize most potential pathogens and mount an immune response, preventing diseases (Jones and Dangl, 2006; Jones *et al*., 2016). Receptors on the plant cell surface can recognize elicitor molecules, which are often widely conserved among major groups of microbes (Jones and Dangl, 2006) and termed pathogen‐associated molecular patterns (PAMPs). PAMPs activate an immune response known as PAMP‐triggered immunity (PTI). However, many plant pathogens produce effector proteins which can disrupt PTI signalling, allowing an infection to be established. In turn, plants employ Resistance (R) proteins, which provide a second layer of pathogen detection by perceiving the intracellular effector proteins or their effects in the cell. Most R proteins belong to the nucleotide‐binding leucine‐rich repeat (NLR) family (Maekawa *et al*., 2011).

NLR activation leads to rapid and strong defence responses, including the production of reactive oxygen species, production of antimicrobial compounds and induction of a type of programmed cell death known as the hypersensitive response (HR). While these defence outputs are quite effective at preventing pathogen spread, they are detrimental to plant growth. Such negative effects are obvious in mutants with mutations that cause constitutive defence signalling, which results in severe dwarfism (Li *et al*., 2001; Wang *et al*., 2013; Yang *et al*., 2010). Thus, in the absence of pathogen attack, negative regulation of NLRs and their downstream signalling components is essential for optimal plant growth.

One important negative regulatory mechanism in eukaryotes is through targeted protein degradation by the ubiquitin proteasome system (UPS). In the UPS pathway, substrates are marked for degradation by the addition of the small protein ubiquitin (Vierstra, 2009). Ubiquitin can be conjugated onto lysine residues within the substrate itself, or onto a previously added ubiquitin moiety, forming a polyubiquitin chain. Polyubiquitinated substrates can subsequently be recognized by the 26S proteasome, a large protein complex with proteolytic function. The 26S proteasome is made up of the 19S regulatory particle, which recognizes the polyubiquitin conjugates and unfolds them, and the 20S core particle (CP), the proteolytic core that possesses multiple subunits with protease activity. The UPS system is critical in regulating almost all biological processes in higher plants, including immunity. Many regulatory proteins in plant defence, including PTI and NLR immune receptors, have been found to be under the control of the UPS system to guarantee an optimum immune output.

The *Mutant, snc1 enhancing* (*MUSE*) screen is a forward genetic screen for mutants that enhances the autoimmunity mediated by a gain‐of‐function NLR mutant *suppressor of npr1-1 constitutive 1* (*snc1*), with the aim of identifying novel negative regulators of immunity (Huang *et al*., 2013). A number of the *muse* mutants identified in this screen are impaired in the turnover of the SNC1 protein by the UPS (Cheng *et al.*, 2011; Copeland *et al*., 2016; Huang *et al*., 2014a, 2014b, 2016; Xu *et al.*, 2015b). For example, a partial loss‐of‐function mutant of the *Arabidopsis thaliana* ATPase, Cell Division Control protein 48 homologue A (AtCDC48A) causes increased SNC1 protein accumulation, which is associated with enhanced disease resistance and dwarf, curly‐leaf morphology (Copeland *et al*., 2016). CDC48 homologues function in UPS‐mediated degradation of diverse substrates and are essential genes in eukaryotes, including *Arabidopsis*, yeast and animals (Baek *et al*., 2013; Dai and Li, 2001; Meyer *et al*., 2012; Rancour *et al*., 2002).

The best characterized function of CDC48 in the UPS is promoting the degradation of substrates by facilitating their recognition by the proteasome. A number of other proteins also assist in UPS‐mediated degradation by shuttling polyubiquitinated substrates to the proteasome, modulating proteasome activity (Yu and Matouschek, 2017). PI31 (also called PSMF1) has been reported to modulate proteasome activity in animal cells. While PI31 was shown to inhibit 20S CP activity *in vitro*, over‐expression of PI31 in the cell appears to increase the activity of the fully assembled 26S proteasome (Clemen *et al*., 2015; Li *et al*., 2014). In *Arabidopsis*, the PI31 homologue PTRE1 also positively regulates cellular proteasome activity (Yang *et al*., 2016). Upon perception of the plant hormone auxin, proteasome activity in wild‐type plants is reduced, while *ptre1* mutants have constitutively lower proteasome activity that is unaffected by auxin. This suggests that a reduction in PTRE1 function is responsible for auxin‐mediated proteasome suppression. As a general regulator of 26S proteasome, it is not surprising that *ptre1* plants also display a dwarf, curly‐leaf morphology similar to many mutants with constitutive immune signalling, including *Atcdc48A‐4* (Fig. 1A) (Copeland *et al*., 2016; Yang *et al*., 2016). This striking resemblance between *ptre1* and *Atcdc48A‐4* plants prompted us to examine whether *PTRE1* also plays a role in immune regulation.

**Figure 1:**
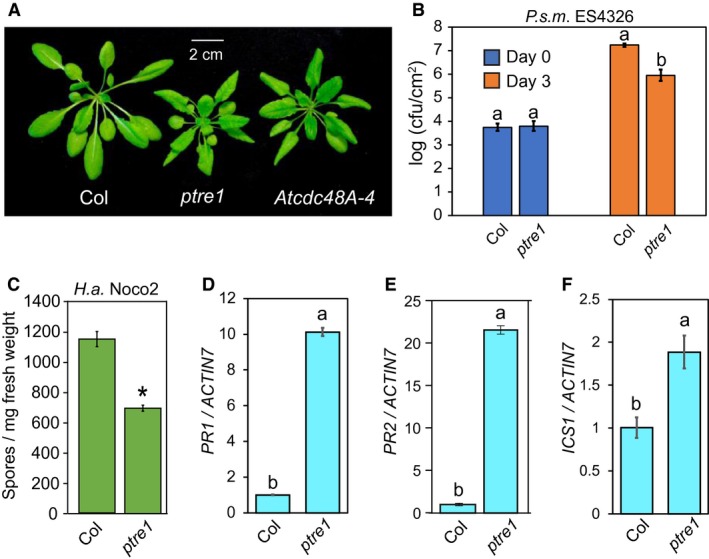
PTRE1 negatively regulates immunity. (A) Morphology of soil‐grown 5‐week‐old *Arabidopsis thaliana* plants of the indicated genotypes. (B) Growth of the bacterial pathogen *Pseudomonas syringae* pv. *maculicola* (*P.s.m.*) ES4326 on plants of the indicated genotypes. Plants were infiltrated with bacterial suspension (OD_600_ = 0.001) and bacterial titre was measured after 0 and 3 days. Error bars represent standard deviation (*n* = 3). The letters indicate significant difference between the different genotypes as determined using a Tukey HSD test. Genotypes denoted using different letters have significant difference (*P* < 0.01). The experiment was performed independently three other times with similar results. (C) Growth of *Hyaloperonospora arabidopsidis* (*H.a*.) Noco2 on plants of the indicated genotypes. Plants were approximately 2.5 weeks old when spray‐inoculated with spore solution of the oomycete (10^5^ spores/mL). Spores were counted 1 week later. Error bars represent standard error of the mean (SEM). The asterisk indicates a significant difference (*P* < 0.05) from Col, as determined by a *t*‐test. (D–F) *PR1* (D), *PR2* (E) and *ICS1* (F) gene expression in the indicated genotypes. qRT‐PCR was performed using RNA extracted from 4‐week‐old plants grown on soil. The letters indicate significant differences between the different samples as determined using a Tukey HSD test. Samples denoted using different letters have a significant difference (*P* < 0.01). The error bars represent standard deviation of the technical replicates (*n* = 3). The experiment was performed two other times with similar results.

In order to determine whether the *ptre1* morphological phenotype is associated with increased immune signalling, we performed infection assays using the virulent bacterial pathogen *Pseudomonas syringae* pv. *maculicola* (*P.s.m.*) ES4326 and an oomycete pathogen *Hyaloperonospora arabidopsidis* (*H.a.*) Noco2 according to the protocols described by Li *et al*. (2001). Enhanced disease resistance was observed when *ptre1* mutant plants were challenged with both pathogens (Fig. 1B,C). We also examined expression of the defence marker genes *PATHOGENESIS RELATED 1* (*PR1*), *PR2* and *ICS1* (*Isochorismate synthase 1*) using qRT‐PCR. *ptre1* plants indeed also showed increased expression of these genes (Fig. 1D–F). Taken together, knocking out *PTRE1* leads to autoimmunity similar to that in *Atcdc48A‐4*. However, the *ptre1* mutant is not as temperature sensitive as the other autoimmune mutants, such as *snc1* or *Atcdc48A‐4* (Fig. S1). This is likely because PTRE1 is a general proteasome regulator and knocking out PTRE1 is expected to yield other growth and developmental defects unrelated to immunity.

*Atcdc48A‐4* and a number of other *muse* mutants have reduced turnover of NLR proteins such as SNC1, causing constitutive immune signalling. Since PTRE1 is thought to promote protein turnover by the proteasome, and may interact with AtCDC48A (Clemen *et al.*, 2015), we first tested whether *ptre1* mutants exhibit altered SNC1 levels. Total protein was extracted according to Huang *et al.* (2014b), and immunoblotted using a SNC1‐specific antibody (Li *et al.*, 2010). SNC1 protein abundance was significantly higher in *ptre1* plants than in the wild‐type (WT) (Fig. 2A,B). We did not observe increased *SNC1* gene expression in the *ptre1* plants, suggesting that the increased SNC1 protein abundance is due to increased protein stability (Fig. 2C). To further confirm that the increase in SNC1 protein level is not due to indirect effects of increased immunity in the *ptre1* mutant, we introduced the *ptre1* mutation into the *eds1* background, which is impaired in feedback up‐regulation of NLR gene transcription (Wiermer *et al*., 2005). The *ptre1 eds1* double mutant was morphologically similar to the *ptre1* single mutant (Fig. 2D), likely due to pleiotropic effects such as the auxin‐related phenotypes reported by Yang *et al*. (2016). A significant increase in SNC1 protein abundance was still observed in the *ptre1 eds1* double mutant plants, further supporting our hypothesis that PTRE1 promotes SNC1 turnover (Fig. 2E,F).

**Figure 2:**
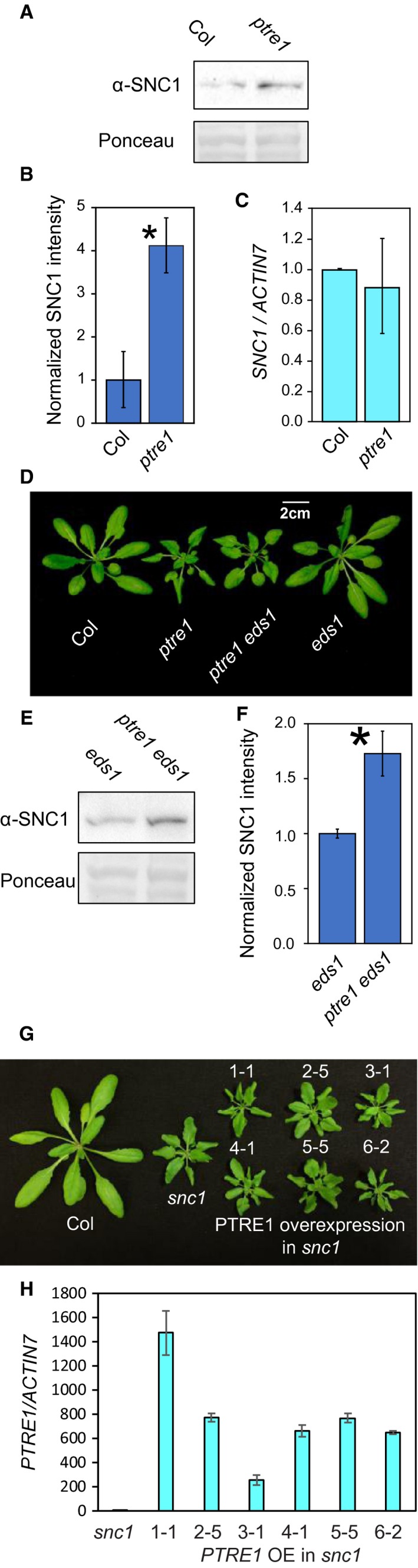
Knockout mutant of *PTRE1* displays increased SNC1 protein stability. (A) SNC1 protein level in the indicated genotypes. Total protein was extracted from 3‐week‐old *Arabidopsis thaliana* plants grown on soil and immunoblot analysis was performed using a SNC1‐specific antibody (Li *et al*., 2010). Ponceau staining is shown as a loading control. (B) Quantification of SNC1 protein level from repeats of experiments as described in (A). For each sample, the intensity of the SNC1 band was normalized to the Ponceau staining, then the relative intensity in Col was set to 1. Error bars represent standard error of the mean. Asterisks indicate a significant difference (*P* < 0.05) from Col as determined by a *t*‐test. The experiment was repeated at least three times with similar results. (C) *SNC1* gene expression in the indicated genotypes. qPCR was performed on RNA extracted from plants in (A). *SNC1* expression was normalized to *ACTIN7*. Error bars represent standard error of the mean. (D) Morphology of 4‐week‐old soil‐grown plants of the indicated genotypes. (E) SNC1 protein level in the indicated genotypes. Experiment was performed as in (A). (F) Quantification of SNC1 protein level from the repeats of experiments as described in (E). (G) Morphology of *snc1* mutants over‐expressing PTRE1. *PTRE1* driven by the 35S promoter was introduced into the *snc1* background. The image shows plants from six independent single copy transgenic lines. (H) *PTRE1* expression levels in the *PTRE1 * over‐expression (OE) lines in the *snc1* background (as in G)*.* Homozygous T3 plants were used for the *PTRE1* expression level quantification. qRT‐PCR was performed using RNA extracted from 4‐week‐old plants grown on soil. The *PTRE1* expression level is set to 1 for *snc1*. The error bars represent standard deviation of the technical replicates (*n* = 3).

As PTRE1 negatively regulates the level of SNC1 protein, we wondered if the levels of other NLRs are also affected. Therefore, we introduced the *ptre1* mutation, by crossing, into lines expressing either RPM1‐myc (Boyes *et al*., 1998) or RPS4‐HA (Wirthmueller *et al*., 2007). Neither NLR showed increased abundance in the *ptre1* background compared to the parental line (Fig. S2A). While it is possible that loss of *PTRE1* causes a change in RPM1 or RPS4 levels that is undetectable in an immunoblot, this result suggests that PTRE1 displays some levels of specificity in regulating protein turnover, even within the NLR family.

The exact function of PTRE1 in proteasome regulation is still unclear (Fort *et al*., 2015). Some genetic and biochemical evidence indicates that *PTRE1* homologues stimulate 26S proteasome activity. Over‐expression of *Drosophila* PI31 suppresses phenotypes caused by mutant proteasome subunits with impaired function (Bader *et al*., 2011). Yang *et al*. (2016) reported that over‐expression of PTRE1 in *Arabidopsis* resulted in reduced accumulation of IAA proteins. Therefore, we tested whether PTRE1 over‐expression could decrease SNC1 stability. A *35S::PTRE1* over‐expression construct was made and *35S::PTRE1* could complement the *ptre1* mutant morphological phenotype and bring down the SNC1 protein level to near wild‐type level (Fig. S2B,C), suggesting that *35S::PTRE1* is functional. Since wild‐type plants show a low steady‐state SNC1 level, over‐expression of PTRE1 would be unlikely to cause an observable protein level reduction. Instead, we introduced a *35S::PTRE1* over‐expression construct into the *snc1* mutant, in which the dwarf morphology and increased immunity are due to increased SNC1 protein abundance (Cheng *et al.*, 2011). If PTRE1 over‐expression reduces SNC1 stability, we would expect to see full or subtle suppression of the autoimmune phenotype in *35S::PTRE1 snc1* plants. The *PTRE1* expression level in the over‐expression lines was quantified by qRT‐PCR and *PTRE1* was found to be significantly over‐expressed in these transgenic lines (Fig. 2F). However, none of these lines showed any obvious morphological differences from the *snc1* single mutant, suggesting that SNC1 turnover is not affected upon PTRE1 over‐expression (Fig. 2G). It is possible that PTRE1 is not the rate‐limiting component in the degradation of SNC1. Similarly, no general effect was seen on the degradation of known UPS targets when PI31 was over‐expressed in mammalian cells (Li *et al*., 2014; Zaiss *et al*., 2002).

The *ptre1 eds1* was further studied and its immune phenotype was characterized. In an infection assay using *P.s.m*. ES4326, the bacterial growth in the *ptre1 eds1* double mutant was comparable to the *eds1* single mutant (Fig. 3A). This further shows that the increased resistance of *ptre1* is due to immunity‐related changes rather than indirect non‐specific pleiotropic effects of the *ptre1* mutation. Consistently, *PR1*, *PR2* and *ICS1* gene expression levels are lower in the *ptre1 eds1* double mutant compared to the *ptre1* single mutant (Fig. 3B–D).

**Figure 3:**
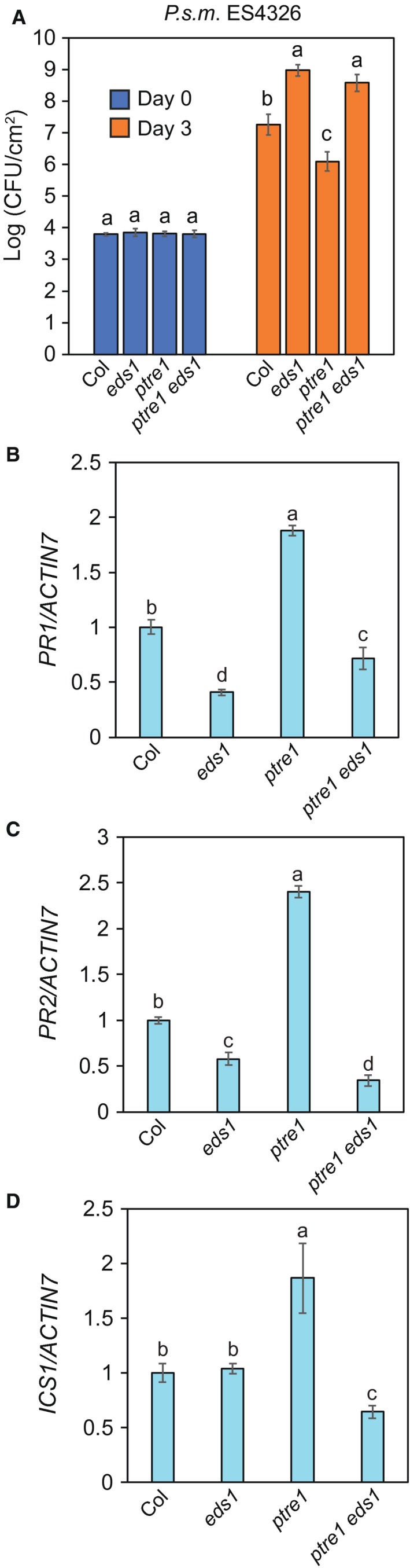
*eds1* suppresses the enhanced immunity of the *ptre1* mutant. (A) Growth of the bacterial pathogen *Pseudomonas syringae* pv. *maculicola* (*P.s.m*.) ES4326 in leaves of *Arabidopsis thaliana* plants of the indicated genotypes. Plants were infiltrated with bacterial suspension (OD_600_ = 0.001) and bacterial titre was measured after 0 and 3 days. Error bars represent sample standard deviation (*n* > 2). The letters indicate significant difference between the different samples as determined using a Tukey HSD test. Samples denoted using different letters have significant difference (*P* < 0.05). The experiment was performed independently three other times with similar results. (B–D) *PR1* (B), *PR2* (C) and *ICS1* (D) gene expression in the indicated genotypes. qRT‐PCR was performed using RNA extracted from 4‐week‐old plants grown on soil. The letters indicate significant difference between the different samples as determined using a Tukey HSD test. Samples denoted using different letters have significant difference (*P* < 0.01). The error bars represent standard deviation of the technical replicates (*n* = 3). The experiment was performed two other times with similar results.

The molecular mechanism of PTRE1 function remains to be elucidated. PI31 interacts with F‐box proteins in both *Drosophila* and humans (Bader *et al*., 2011; Kirk *et al.*, 2008). However, at least in *Drosophila* this interaction seems to stabilize PI31 but is not strictly required for its proteasome‐regulatory function. In addition, the murine homologues of AtCDC48A and PTRE1 (VCP and PMSF1, respectively) show a direct interaction *in vitro*, which modulates their ability to regulate the activity of purified 20S proteasome core particles (Clemen *et al*., 2015). Since *Arabidopsis* PTRE1 and AtCDC48A are both thought to positively regulate 26S proteasome activity, we hypothesized that they may also interact *in planta*. Therefore, we tested for protein–protein interactions using a co‐immunoprecipitation (co‐IP) assay, which was carried out as described by Xu *et al*. (2015a). PTRE1 fused to a C‐terminal flag epitope tag (PTRE1‐flag) and AtCDC48A with a C terminal GFP tag (AtCDC48A‐GFP) (Copeland *et al*., 2016) were transiently co‐expressed in *Nicotiana* *benthamiana* using *Agrobacterium*‐mediated transformation at an OD_600_ of 0.3 for each strain, with a strain containing an empty vector being used as negative control. Infiltrated leaf tissue was harvested after 48 h, and α‐flag conjugated to agarose beads was used for the co‐IP. As shown in Fig. 4A, AtCDC48A‐GFP did not co‐IP with PTRE1‐flag. However, it is still possible that PTRE1 may work together with AtCDC48A through a weak or transient association that cannot be detected by co‐IP.

**Figure 4:**
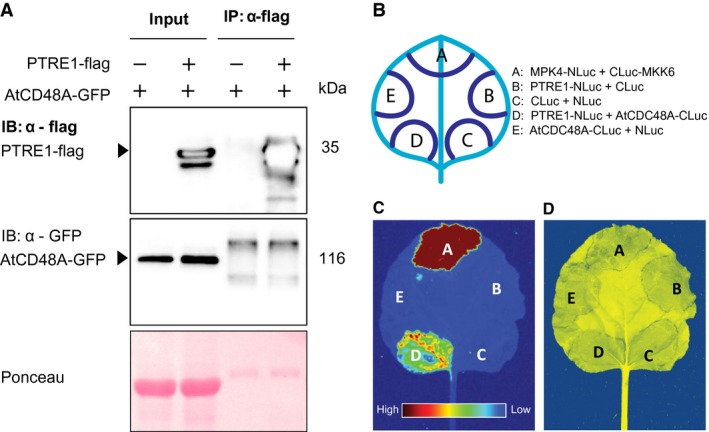
PTRE1 does not co‐immunoprecipitate with AtCDC48A but interacts with it in the split luciferase assay. (A) AtCDC48A‐GFP was co‐expressed in *Nicotiana benthamiana* leaves with either PTRE1‐flag or with an empty vector, and the total protein was extracted after 48 h. The protein was subjected to immunoblot analysis using the indicated antibodies. Arrowheads show the size of the prospective bands and the approximate protein sizes are indicated to the right of the blots. Ponceau staining of the blot is shown as a loading control. (B) Cartoon showing the infiltration areas on the *N. benthamiana* leaf for the split luciferase assay. The legend denotes the gene constructs in the *Agrobacterium* infiltrated in that area. (C) Luminescence recorded after infiltration of 1 mM luciferin into the *Agrobacterium* infiltration sites. The heat map scale is on the bottom with dark red representing high levels of luminescence and blue representing low levels of luminescence. (D) False colour image of the same leaf photographed under white light showing the infiltration areas.

The interaction between the two proteins was further examined using the split luciferase complementation assay. *PTRE1* and *AtCDC48A* were cloned into a modified pCambia1300 vector with the CaMV 35S promoter. *PTRE1* was fused to the N‐terminal part of the luciferase protein at its C terminus (PTRE1‐NLuc). *AtCDC48A* was fused to the C‐terminal fragment of luciferase at its C terminus (AtCDC48‐CLuc). These constructs were then transformed into *Agrobacterium* strain GV3101 for infiltration into the leaves of *N. benthamiana*. The *Agrobacterium* strains carrying different vectors were infiltrated using a syringe into the leaves in combinations as seen in Fig. 4B with each of the *Agrobacterium* strains diluted to a final OD_600_ of 0.2 with the same infiltration solution as used in the co‐IP experiment. The MPK4‐NLuc and CLuc‐MKK6 interaction serves as a positive control. Luminescence was observed in the areas of the leaf infiltrated with the positive control and the area infiltrated with PTRE1‐NLuc and AtCDC48‐CLuc (Fig. 4C,D), demonstrating a direct protein–protein interaction between the two proteins. The reason we did not see interaction in co‐IP but observed interaction in the split luciferase assay could be because the two proteins have a weak and/or transient interaction that is difficult to observe with co‐IP. Overall, a more thorough examination of the genetic and biochemical interaction network of PTRE1 is required to understand the mechanism by which it regulates proteasome activity.

A BLAST search of the *Arabidopsis* genome using *PTRE1* as a query revealed a homologous sequence, AT1G48530, which we named *PTRE1h*. Based on an alignment of *PTRE1* and *PTRE1h*, *PTRE1h* is a truncated sequence, containing homology to the first three exons of *PTRE1* (Fig. S3A). However, the deduced amino acid sequences of both genes within this region share 56% identity and 70% similarity, prompting us to test whether *PTRE1h* may share redundant function with *PTRE1*. A *ptre1h* mutant containing an exonic T‐DNA insertion was morphologically indistinguishable from Col (Fig. S3B). To test for possible redundancy between *PTRE1* and *PTRE1h*, a *ptre1 ptre1h* double mutant was generated. The morphological phenotype of the double mutant was not altered compared to the *ptre1* single mutant, nor were double mutants more resistant to *H.a.* Noco2 or *P.s.m* ES4326 infection (Fig. S3C,D). However, defence markers *PR1*, *PR2* and the *ICS1* expression levels are consistently higher in the *ptre1 ptre1h* double mutant as compared to those in the *ptre1* single mutant (Fig. S3E–G), suggesting that PTRE1h is partially redundant with PTRE1. The difference in immunity between *ptre1* and *ptre1 ptre1h* could be too small to be detected using the infection experiments that we performed. When the SNC1 protein level was quantified in the *ptre1* and *ptre1 ptre1h* mutants to see whether the *ptre1h* mutation further contributed to SNC1 protein stability, we could observe an overall increase in the SNC1 protein level in the *ptre1 ptre1h* double mutant compared to the single mutant (Fig. S4A). However, this difference was not statistically significant among repeats of the experiment (Fig. S4B).

NLR turnover through the UPS is crucial to limit NLR over‐accumulation and prevent autoimmunity (Holt *et al*., 2005; Li *et al*., 2015). In this study, we show that the proteasome regulator *PTRE1* is a negative regulator of immunity and promotes SNC1 turnover. The *ptre1* mutant displays a dwarf phenotype, increased defence marker gene expression and enhanced resistance to virulent pathogens (Fig. 1). SNC1 protein abundance is also increased in the *ptre1* mutant. The *Arabidopsis* genome contains a truncated homologue of *PTRE1*, *PTRE1h*, that shares high homology in the N‐terminal region (Fig. S3). PTRE1h seems to be partially redundant with PTRE1.

The *ptre1* mutant displays a range of defects potentially related to auxin, abscisic acid and brassinosteroid signalling, suggesting that PTRE1 promotes the turnover of many different substrates (Yang *et al*., 2016). Our finding in this study that PTRE1 is involved in SNC1 turnover adds to the previously identified model of NLR degradation by identifying another component that likely acts together with the proteasome and AtCDC48A. However, we still do not know the molecular events involved in PTRE1 regulation of proteasome activity. A more thorough characterization of protein interactors of PTRE1 will be required to determine how it facilitates the degradation of specific substrates.

## Supporting information


**Fig. S1** *ptre1 *morphological phenotype is not suppressed when grown at high temperature. The figure shows the plants of the indicated genotypes grown at the indicated temperatures.

**Fig. S2** PTRE1 does not affect RPM1 and RPS4 levels, and PTRE1 over‐expression is able to complement the phenotype of the *ptre1 *mutant. (A) Protein levels of RPM1‐myc (left) and RPS4‐HA (right) in the indicated genotypes. Total protein was extracted from 4‐week‐old plants grown on soil, and immunoblot analysis was performed using the respective antibodies. Ponceau staining of the blot is shown as a loading control. (B) Two T1 *ptre1 *plants (#1 and #2) over‐expressing PTRE1 are shown above with *ptre1 *and Col as controls. (C) SNC1 protein level in the complementation lines shown above. The Ponceau‐stained band is shown as the loading control.

**Fig. S3** PTRE1h is partially redundant with PTRE1. (A) Gene model of *PTRE1 *and *PTRE1h*. Exons are represented as boxes and introns are represented as lines. Arrowheads indicate the positions of the T‐DNA insertions in the mutants described. (B) Morphology of 5‐week‐old soil‐grown plants of the indicated genotypes. (C) Growth of *Hyaloperonospora arabidopsidis. *Noco2 on plants of the indicated genotypes. Three‐week‐old plants were inoculated with spore suspension (10^5^  spores/mL) and grown at 18 °C for a week. Spores were counted 1 week later. Error bars represent sample standard deviation (*n* = 3). The letters indicate significant difference between the different samples as determined using a Tukey Honest Significant Difference (HSD) test. Samples denoted using different letters have significant difference (*P* < 0.05). The experiment was performed independently two other times with similar results. (D) Growth of the bacterial pathogen *Pseudomonas syringae *pv. *maculicola* ES4326 in leaves of plants of the indicated genotypes. Plants were infiltrated with bacterial suspension (OD_600_ = 0.001) and bacterial titre was measured after 0 and 3 days. Error bars represent sample standard deviation (*n* = 3). The letters indicate significant difference between the different samples as determined using a Tukey HSD test. Samples denoted using different letters have significant difference (*P* < 0.01). The experiment was performed independently three other times with similar results. (E–G) *PR1 *(E), *PR2 *(F) and *ICS1 *(G) gene expression in the indicated genotypes. qRT‐PCR was performed using RNA extracted from 4‐week‐old plants grown on soil. The letters indicate significant difference between the different samples as determined using a Tukey HSD test. Samples denoted using different letters have significant difference (*P* < 0.01). The experiment was performed two other times with similar results.

**Fig. S4** SNC1 protein levels in the *ptre1 *and the *ptre1 ptre1h *are not significantly different. (A) SNC1 protein levels quantified in 4‐week‐old soil‐grown *Arabidopsis thaliana* plants of the indicated genotypes using a SNC1‐specific antibody (Li *et al.*, 2010). The Ponceau‐stained band is shown as a loading control. The experiment was repeated three times with similar results. (B) The SNC1 protein band intensities were quantified using ImageJ, normalized to the loading control band and then the relative intensity in Col was set to 1. The bar graph shows the mean normalized SNC1 band intensity of the three repeats of the experiment and the error bars denote the standard deviation of the normalized band intensity between experiments. The letters indicate the significant difference between the different samples as determined using a Tukey HSD test. Samples denoted using different letters have significant difference (*P* < 0.01).

**Method** Plant growth condition and pathogen infection assays; quantitative RT‐PCR; protein extraction and immunoprecipitation.
