# Supplementary material for: The proteasome regulator PTRE1 contributes to the turnover of SNC1 immune receptor
Source: Mol Plant Pathol. 2019 Aug 8;20(11):1566–73. doi: 10.1111/mpp.12855 (PMC6804346; doi:10.1111/mpp.12855)
Supplement: Supplementary file 1 — Fig. S1 ptre1 morphological phenotype is not suppressed when grown at high temperature. The figure shows the plants of the indicated genotypes grown at the indicated temperatures. [file MPP-20-1566-s001.pdf]

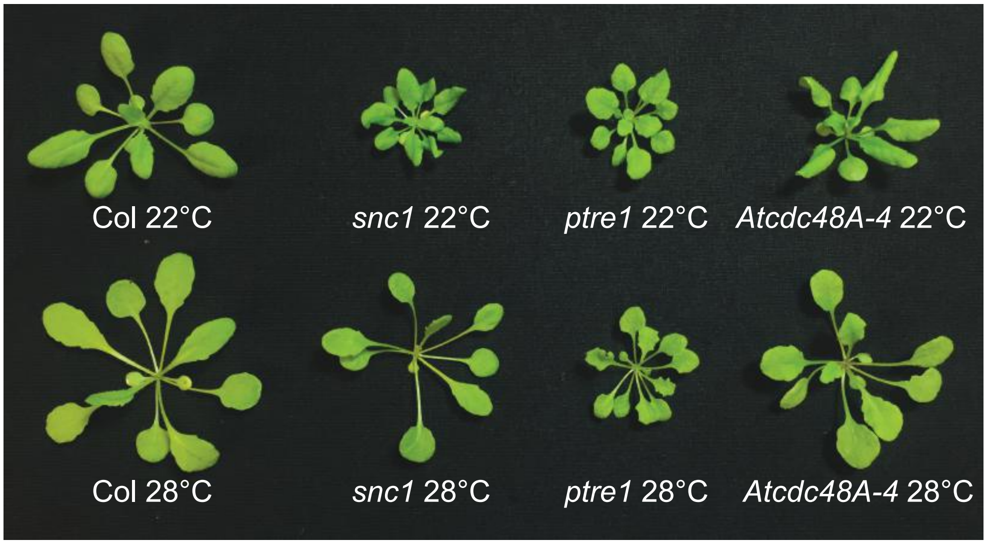

Figure S1. ***ptre1*** **morphological phenotype is not suppressed when grown at high temperature**

The figure shows the plants of the indicated genotypes grown at the indicated temperatures.
