# Supplementary material for: The proteasome regulator PTRE1 contributes to the turnover of SNC1 immune receptor
Source: Mol Plant Pathol. 2019 Aug 8;20(11):1566–73. doi: 10.1111/mpp.12855 (PMC6804346; doi:10.1111/mpp.12855)
Supplement: Supplementary file 2 — Fig. S2 PTRE1 does not affect RPM1 and RPS4 levels, and PTRE1 over‐expression is able to complement the phenotype of the ptre1 mutant. (A) Protein levels of RPM1‐myc (left) and RPS4‐HA (right) in the indicated genotypes. Total protein was extracted from 4‐week‐old plants grown on soil, and immunoblot analysis was performed using the respective antibodies. Ponceau staining of the blot is shown as a loading control. (B) Two T1 ptre1 plants (#1 and #2) over‐expressing PTRE1 are shown above with ptre1 and Col as controls. (C) SNC1 protein level in the complementation lines shown above. The Ponceau‐stained band is shown as the loading control. [file MPP-20-1566-s002.pdf]

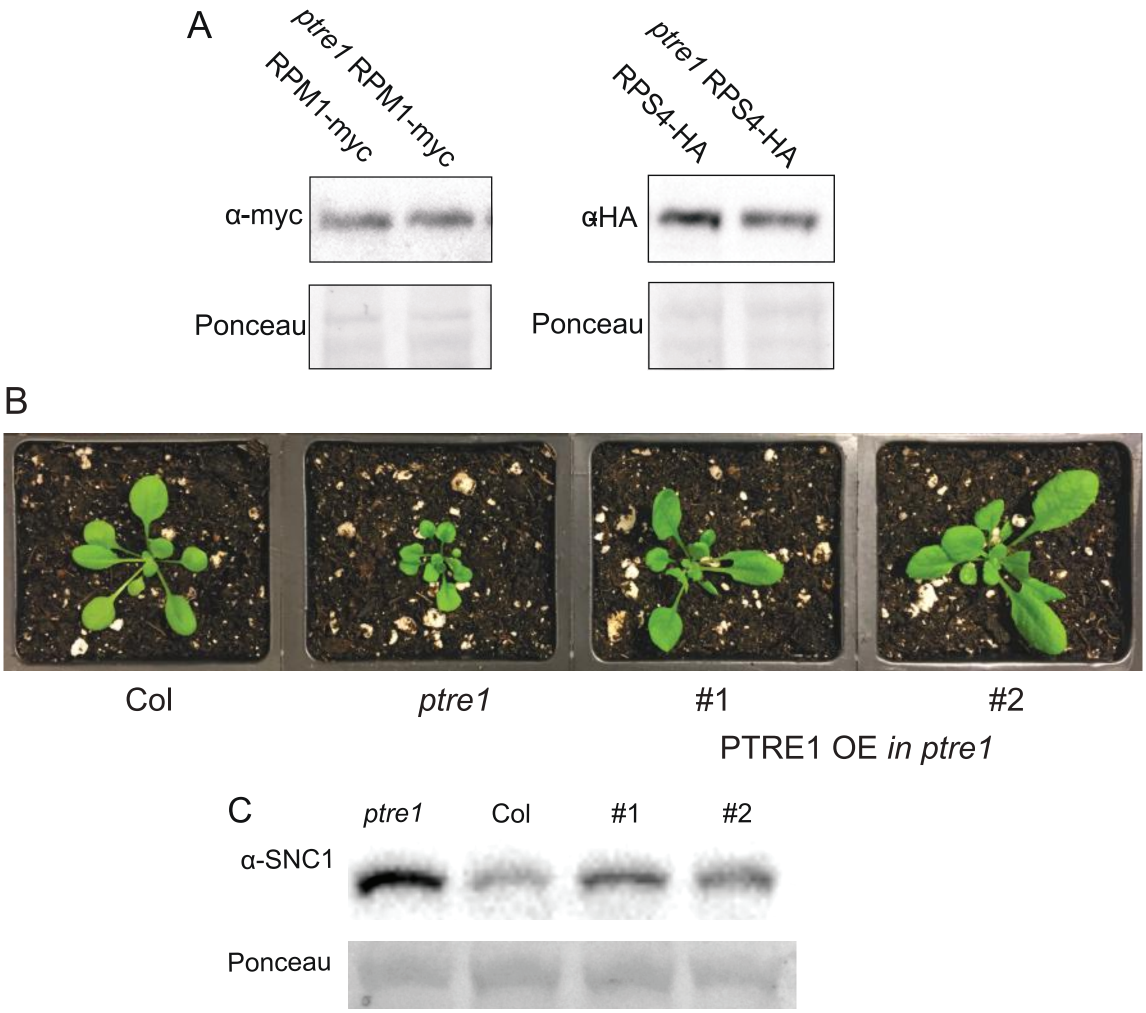

Figure S2. PTRE1 does not affect RPM1 and RPS4 levels and PTRE1 OE is able to complement the phenotype of the *ptre1* mutant.

(A) Protein levels of RPM1-myc (left) and RPS4-HA (right) in the indicated genotypes. Total protein was extracted from 4-week-old plants grown on soil, and immunoblot analysis was performed using the respective antibodies. Ponceau staining of the blot is shown as a loading control.

(B) Two T1 *ptre1* plants (#1 and #2) overexpressing PTRE1 are shown above with *ptre1* and Col as controls.

(C) SNC1 protein level in the complementation lines shown above. The ponceau band is shown as the loading control.
