# Supplementary material for: The proteasome regulator PTRE1 contributes to the turnover of SNC1 immune receptor
Source: Mol Plant Pathol. 2019 Aug 8;20(11):1566–73. doi: 10.1111/mpp.12855 (PMC6804346; doi:10.1111/mpp.12855)
Supplement: Supplementary file 3 — Fig. S3 PTRE1h is partially redundant with PTRE1. (A) Gene model of PTRE1 and PTRE1h. Exons are represented as boxes and introns are represented as lines. Arrowheads indicate the positions of the T‐DNA insertions in the mutants described. (B) Morphology of 5‐week‐old soil‐grown plants of the indicated genotypes. (C) Growth of Hyaloperonospora arabidopsidis. Noco2 on plants of the indicated genotypes. Three‐week‐old plants were inoculated with spore suspension (105 spores/mL) and grown at 18 °C for a week. Spores were counted 1 week later. Error bars represent sample standard deviation (n = 3). The letters indicate significant difference between the different samples as determined using a Tukey Honest Significant Difference (HSD) test. Samples denoted using different letters have significant difference (P < 0.05). The experiment was performed independently two other times with similar results. (D) Growth of the bacterial pathogen Pseudomonas syringae pv. maculicola ES4326 in leaves of plants of the indicated genotypes. Plants were infiltrated with bacterial suspension (OD600 = 0.001) and bacterial titre was measured after 0 and 3 days. Error bars represent sample standard deviation (n = 3). The letters indicate significant difference between the different samples as determined using a Tukey HSD test. Samples denoted using different letters have significant difference (P < 0.01). The experiment was performed independently three other times with similar results. (E–G) PR1 (E), PR2 (F) and ICS1 (G) gene expression in the indicated genotypes. qRT‐PCR was performed using RNA extracted from 4‐week‐old plants grown on soil. The letters indicate significant difference between the different samples as determined using a Tukey HSD test. Samples denoted using different letters have significant difference (P < 0.01). The experiment was performed two other times with similar results. [file MPP-20-1566-s003.pdf]

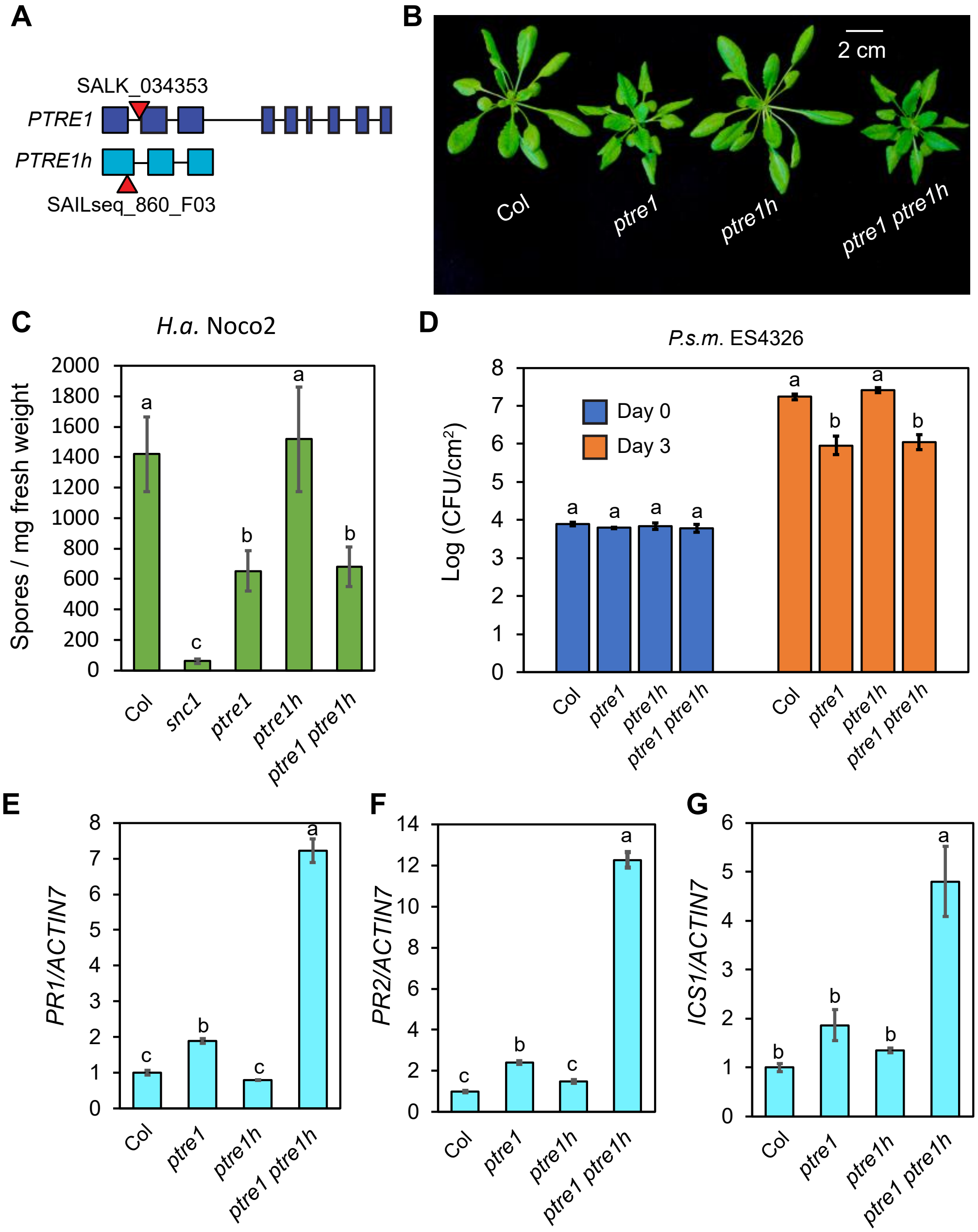


**Figure S3. PTRE1h is partially redundant with PTRE1.**

(A) Gene model of *PTRE1* and *PTRE1h*. Exons are represented as boxes and introns are represented as lines. Arrowheads indicate the positions of the T-DNA insertions in the mutants described.

(B) Morphology of 5-week-old soil-grown plants of the indicated genotypes.

(C) Growth of *H.a.* Noco2 on plants of the indicated genotypes. 3-week-old plants were inoculated with spore suspension ($10^5$ spores / ml) and grown at 18°C for a week. Spores were counted one week later. Error bars represent sample standard deviation (n=3). The letters indicate significant difference between the different samples as determined using a Tukey HSD (Honest Significant Difference) test. Samples denoted using different letters have significant difference ($p<0.05$). The experiment was performed independently two other times with similar results.

(D) Growth of the bacterial pathogen *P.s.m.* ES4326 in leaves of plants of the indicated genotypes. Plants were infiltrated with bacterial suspension ($OD_{600}$ = 0.001), and bacterial titer was measured after 0 and 3 days. Error bars represent sample standard deviation (n=3). The letters indicate significant difference between the different samples as determined using a Tukey HSD (Honest Significant Difference) test. Samples denoted using different letters have significant difference ($p<0.01$). The experiment was performed independently three other times with similar results.

(E-G) *PR1* (E), *PR2* (F) and *ICS1* (G) gene expression in the indicated genotypes. qRT-PCR was performed using RNA extracted from 4-week-old plants grown on soil. The letters indicate significant difference between the different samples as determined using a Tukey HSD (Honest Significant Difference) test. Samples denoted using different letters have significant difference ($p<0.01$). The experiment was performed two other times with similar results.
