# Supplementary material for: The proteasome regulator PTRE1 contributes to the turnover of SNC1 immune receptor
Source: Mol Plant Pathol. 2019 Aug 8;20(11):1566–73. doi: 10.1111/mpp.12855 (PMC6804346; doi:10.1111/mpp.12855)
Supplement: Supplementary file 4 — Fig. S4 SNC1 protein levels in the ptre1 and the ptre1 ptre1h are not significantly different. (A) SNC1 protein levels quantified in 4‐week‐old soil‐grown Arabidopsis thaliana plants of the indicated genotypes using a SNC1‐specific antibody (Li et al., 2010). The Ponceau‐stained band is shown as a loading control. The experiment was repeated three times with similar results. (B) The SNC1 protein band intensities were quantified using ImageJ, normalized to the loading control band and then the relative intensity in Col was set to 1. The bar graph shows the mean normalized SNC1 band intensity of the three repeats of the experiment and the error bars denote the standard deviation of the normalized band intensity between experiments. The letters indicate the significant difference between the different samples as determined using a Tukey HSD test. Samples denoted using different letters have significant difference (P < 0.01). [file MPP-20-1566-s004.pdf]

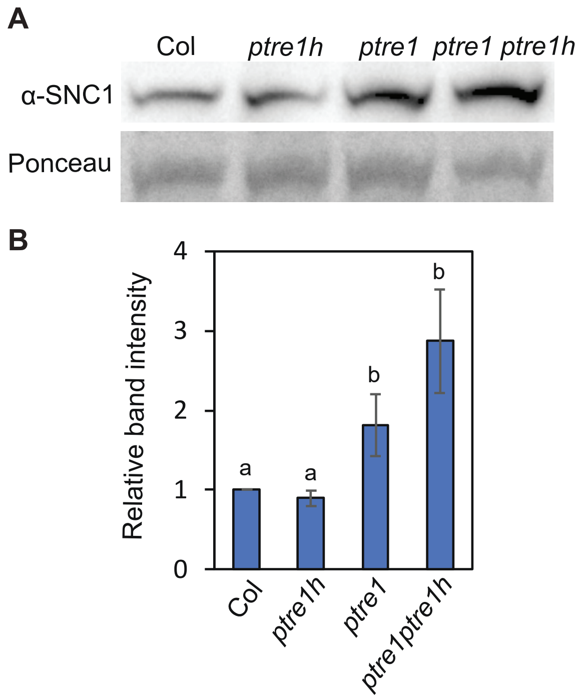

Figure S4. SNC1 protein levels in the *ptre1* and the *ptre1ptre1h* are not significantly different.

(A) SNC1 protein levels quantified in four-week-old soil grown plants of the indicated genotypes using a SNC1 specific antibody (Li et al., 2010). The ponceau band is shown as a loading control. The experiment was repeated three times with similar results.

(B) The SNC1 protein band intensities were quantified using ImageJ, normalized to the loading control band and then the relative intensity in Col was set to 1. The bar graph shows the mean normalized SNC1 band intensity of the three repeats of the experiment and the error bars denote the standard deviation of the normalized band intensity between experiments. The letters indicate significant difference between the different samples as determined using a Tukey HSD (Honest Significant Difference) test. Samples denoted using different letters have significant difference ($p<0.01$).
