# Supplementary material for: The proteasome regulator PTRE1 contributes to the turnover of SNC1 immune receptor
Source: Mol Plant Pathol. 2019 Aug 8;20(11):1566–73. doi: 10.1111/mpp.12855 (PMC6804346; doi:10.1111/mpp.12855)
Supplement: Supplementary file 5 — Method Plant growth condition and pathogen infection assays; quantitative RT‐PCR; protein extraction and immunoprecipitation. [file MPP-20-1566-s005.docx]

**Supplementary Methods**

**Plant growth condition and pathogen infection assays**

Unless specified, both Arabidopsis and tobacco plants were grown in growth rooms at 22ºC under 16-h-day/8-h-night cycles. For *H.a.* Noco2 oomycete infections, one-week-old plate (Standard ½ MS, 1% sucrose, 0.3% Phytagel) germinated plants were transplanted to soil and allowed to grow for 10 more days . They were then spray-inoculated with conidiospores suspended in water at the indicated concentrations. Plants were grown in a chamber at 18°C under 12-h-day/12-h-night cycles for seven days prior to quantification of spores using a haemocytometer.

For *P.s.m.* ES4326 bacterial infections, about four-week-old soil-grown plants were infiltrated with the virulent bacterial pathogen at a concentration of OD_600_=0.001. At zero and three days after infiltration, leaf discs were collected from the plants and the bacterial growth was quantified by serial dilution and plating.

**Quantitative RT-PCR**

About 40 mg of leaf tissue was collected from soil-grown plants and subjected to RNA extraction using an EZ-10 Spin Column kit (BioBasic, Markham, Canada), and 1 µg RNA was reverse transcribed into cDNA following the manufacturer’s protocol (EasyScript^TM^ cDNA Synthesis Kit, Abm). RT-PCR was performed as decribed previously (Zhang et al., 2003).

**Protein extraction and immunoprecipitation**

Transient expression in *N. benthamiana* was carried out as described previously (Wu et al., 2017). About 2.5 g *N. benthamiana* leaves expressing the indicated proteins were ground into powder with liquid nitrogen. The FLAG-tagged-tagged proteins were immunoprecipitated using 20 µl M2 beads (Sigma). Immunoblot analysis was described previously (Wu et al., 2017).

**Supplementary references:**

**Wu Z, Huang S, Zhang X, Wu D, Xia S, Li X** (2017) Regulation of plant immune receptor accumulation through translational repression by a glycine-tyrosine-phenylalanine (GYF) domain protein. Elife **6**

**Zhang Y, Goritschnig S, Dong X, Li X** (2003) A gain-of-function mutation in a plant disease resistance gene leads to constitutive activation of downstream signal transduction pathways in suppressor of npr1-1, constitutive 1. Plant Cell **15:** 2636-2646
